# Update of Spectroscopic Data for 4-Hydroxydictyolactone and Dictyol E Isolated from a *Halimeda stuposa* — *Dictyota* sp. Assemblage ^†^

**DOI:** 10.3390/molecules17032929

**Published:** 2012-03-08

**Authors:** Simon P. B. Ovenden, Jonathan L. Nielson, Catherine H. Liptrot, Richard H. Willis, Dianne M. Tapiolas, Anthony D. Wright, Cherie A. Motti

**Affiliations:** Australian Institute of Marine Science, PMB no. 3, Townsville MC, Townsville, 4810, Australia; Email: r.willis@aims.gov.au (R.H.W.); d.tapiolas@aims.gov.au (D.M.T.)

**Keywords:** *Dictyota*, Dictyotaceae, *Halimeda stuposa*, anti-cancer activity, xenicanes, diterpenes, 4-hydroxydictyolactone, dictyol E, 8α,11-dihydroxypachydictyol A, indole-3-carboxaldehyde

## Abstract

The methanol extract of an assemblage of *Halimeda stuposa* and a *Dictyota* sp., yielded three natural products characteristic of *Dictyota* sp., and one of *Halimeda* sp. These included the xenicane diterpene 4-hydroxydictyolactone (**1**), and the diterpenes dictyol E (**2**), 8α,11-dihydroxypachydictyol A (**3**) and indole-3-carboxaldehyde (**4**). A minor revision of **1** and new spectroscopic data for **1** and **2** are provided, along with associated anti-cancer activities of compounds **1**–**4**.

## 1. Introduction

There have been many reports involving chemical investigations of algae from the genera *Halimeda* [1,2,3,4] and *Dictyota* [5,6,7,8,9,10], with *Dictyota* species in particular being a prolific source of novel terpenoids. Considering the well documented history of terpenoid chemistry having significant biological activity [11,12,13], this genus of alga is an attractive target for the discovery of novel bioactive metabolites.

**Figure 1:**
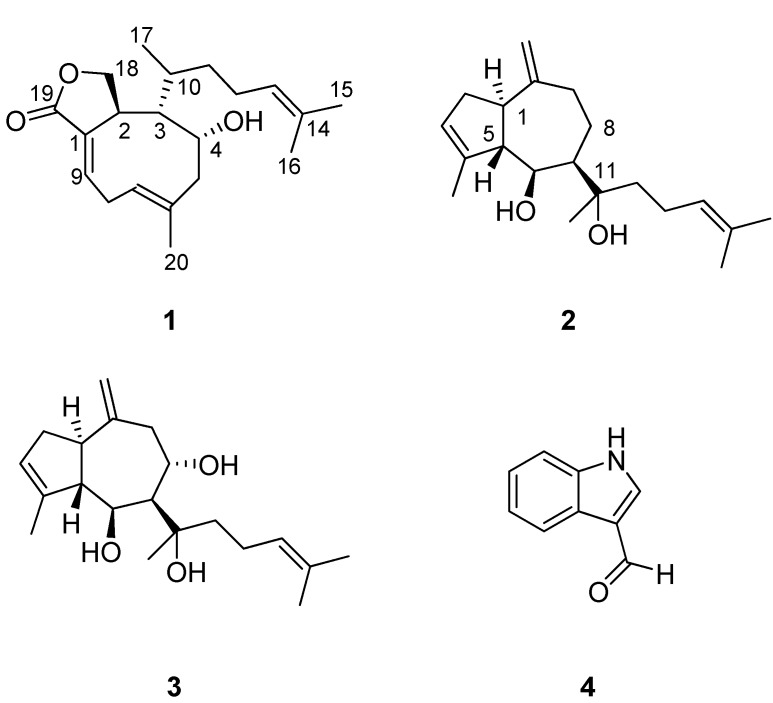
Structures of the xenicane lactone 4-hydroxydictyolactone (**1**), the diterpenes dictyol E (**2**) and 8α,11-dihydroxypachydictyol A (**3**), and indole-3-carboxaldehyde (**4**).

While investigating marine derived extracts for their anti-cancer activity, the ethanol (EtOH) extract of the green-brown alga assemblage of *Halimeda stuposa* and *Dictyota* sp. was found to have significant activity and an unusual profile in the NCI 60 cell line COMPARE analysis [14] The methanol (MeOH) extract of a large scale recollection was subjected to bioassay-guided fractionation, using C_18_ flash vacuum liquid chromatography and preparative C_18_ HPLC, to yield the xenicane lactone 4-hydroxydictyolactone (**1**) [15], as well as the known diterpenes dictyol E (**2**) [16], 8α,11-dihydroxypachydictyol A (**3**) [17], and indole-3-carboxaldehyde (**4**) [18] (Figure 1). Described below are a minor revision of **1**, as well as CD data and molecular modelling studies, in accordance with the absolute configuration previously reported [19], and NMR evidence confirming the presence of the minor *cis* conformer of **1** [20]. Also presented are the complete ^1^H-NMR data for **2**, as well as the biological activities of **1**–**4** against a panel of human tumour and normal mammalian cell lines.

## 2. Results and Discussion

4-Hydroxydictyolactone (**1**) was isolated from the MeOH extract with a HRESIMS molecular weight indicative of the molecular formula C_20_H_30_O_3_ and corresponding to six double bond equivalents. ^1^H- and ^13^C-NMR resonances (Supporting information Table S1) were identical to those first reported for the naturally occurring [15] and the synthetic 4-hydroxy-dictyolactone (**1**) [19], except for the C-7 and C-13 resonances. HSQC correlations (Supporting information Figure S4) were observed from δ_H_ 5.32 (H-7) to δ_C_ 125.3 and from δ_H_ 5.02 (H-13) to δ_C_ 123.9, indicating that the original assignments of these carbons were reversed. The C-1–C-9 double bond was assigned an *E*-configuration owing to the large coupling constants exhibited due to the axial-axial orientation of H-1 (*δ* 5.32, dd, 11.4, 4.2 Hz) and H_a_-2 (*δ* 3.20, dddd, 17.5, 11.4, 2.2, 2.2) [21] whilst ^13^C-NMR data for C-20 (δ_C_ 20.0) confirmed the *E* geometry of C-6–C-7 [22]. All other spectroscopic data matched that reported [15], however, as previously noted by Williams *et al.* [19], a differing optical rotation for the naturally occurring **1** {[α]^21^_D_ −87° (*c* 0.25, CHCl_3_)}was observed. Guella *et al.* [20] showed that **1** undergoes a slow conformation medium-ring flipping between the predominant *trans*- (C-20 *trans* to H-3) and the minor *cis*-conformer (C-20 *cis* to H-3). Further inspection of the ^1^H and COSY NMR data confirmed the presence of the minor *cis*-conformer (Supporting Information Table S1), the ratio of which may influence the optical rotation. Closer inspection of the ^1^H-NMR of the 50% MeOH flash column fraction revealed the presence of both conformers, however, only the *trans*-conformer was detected in the MeOH extract due to overlapping signals and concentration. Molecular modeling studies, where the geometry of both the double bonds (C-2–C-9 and C-6–C-7) in the carbocycle was constrained to *E*, were performed to determine which of the 16 possible stereoisomers (taking into consideration both *trans-* and *cis*-carbocycle conformations giving a total of 32 possibilities) matched the coupling constants observed in the ^1^H-NMR data. As expected four possible structures matched the ^1^H-NMR data, *trans*-2*R*,3*R*,4*S*,10*R*, *trans*-2*R*,3*R*,4*S*,10*S*, *trans*-2*S*,3*S*,4*R*,10*S* and *trans*-2*S*,3*S*,4*R*,10*R*.

**Figure 2:**
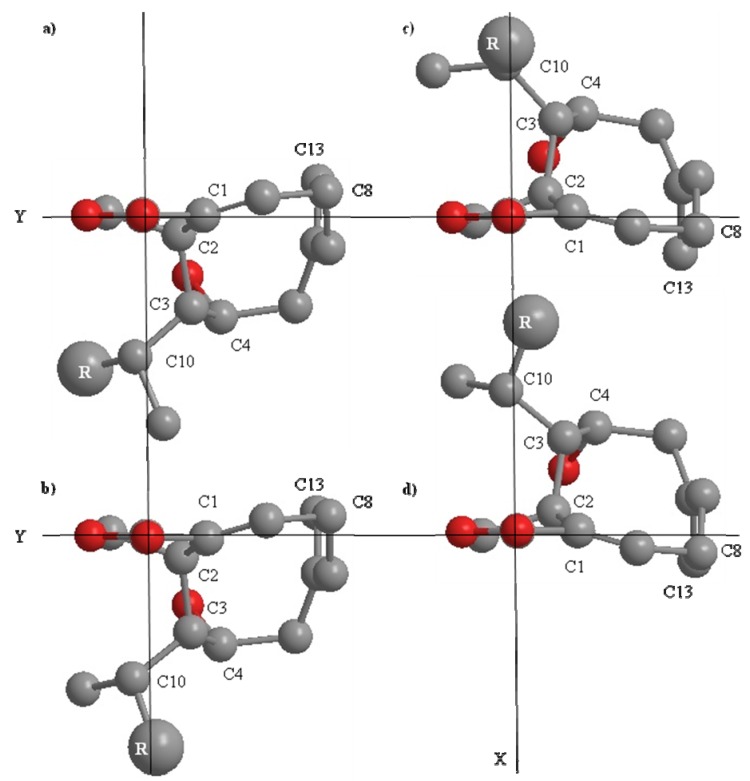
The possible *trans*-conformers, based on ^1^H-NMR coupling constants, viewed along the C=O bond towards C-18, of 4-hydroxydictyolactone (**1**) as obtained from MM2 calculations [25]; (**a**) *trans*-2*R*,3*R*,4*S*,10*R*; (**b**) *trans*-2*R*,3*R*,4*S*,10*S*; (**c**) *trans*-2*S*,3*S*,4*R*,10*S* and (**d**) *trans*-2*S*,3*S*,4*R*,10*R*. R=(CH_2_)_2_CHC(CH_3_)_2_.

The naturally occurring [15,20] and synthetic studies [19] report measurement of optical rotation, but no CD data. The absolute configuration at C-2 of **1** was corroborated by CD measurement. The CD spectrum of **1** showed a large negative Cotton effect at 226 nm (Δε = −38.44, π→π*), and a small positive Cotton effect at 258 nm (Δε = 5.42, n→π*). Applying the quadrant rule [23]; viewing the ring along the O-C-19-C-1 axis, resulted in C-3 extending into the negative upper right quadrant. This finding is reconcilable with an *S* configuration at C-2 (Figure 2c,d) and in agreement with the naturally occurring [15] and the synthetic 4-hydroxydictyolactone (**1**) [19], *trans*-2*S*,3*S*,4*R*,10*R*, where 10*R* has previously been determined by x-ray crystallography [24] and synthetic studies [20].

Dictyol E (**2**) was also isolated from the MeOH extract with a HRESIMS molecular weight indicative of the molecular formula C_20_H_32_O_2_ and corresponding to five double bond equivalents. Initial comparison of experimental ^1^H- and ^13^C-NMR resonances (Table 1) with those reported for the naturally occurring dictyol E (**2**) [16], indicated that the literature ^1^H-NMR data was incomplete and that a full assignment of the structure was required. NMR resonances (Table 1) confirmed the presence of two trisubstituted double bonds (δ_C_ 141.0, 132.0, 124.2, 124.2; δ_H_ 5.34, 1H, br s; 5.16, 1H, br t, *J =* 6.9 Hz) and one disubstituted double bond (δ_C_ 152.0, 107.4; δ_H_ 4.78, 1H, s; δ_H_ 4.76, 1H, s) as well as three olefinic methyls (δ_C_: 25.7, 15.9, 17.5; δ_H_ 1.82, 3H, s; 1.69, 3H, s; 1.62, 3H, s), a tertiary methyl (δ_C_: 25.3; δ_H_ 1.26, 3H, s) and an oxy-methine (δ_C_: 74.4; δ_H_4.20, 1H, dd, *J =* 7.8, 2.0 Hz), consistent with reported values. Five additional methylenes and three methines were also observed.

Analysis of the COSY NMR data for **2** (Table 1) showed an extended ^1^H-^1^H spin system from H-3 (δ_H_ 5.34, 1H, br s) to H_2_-9 (δ_H_ 2.69, 1H, ddd,14.8, 4.6, 2.4 Hz) via H-1 (δ_H_ 2.60, 1H, q, *J =* 9.1 Hz) and H-5 (δ_H_ 2.37, 1H, m), as well as long-range ^4^*J* COSY NMR correlations from H-3 to H_3_-17 and H-5, from H-5 to H_3_-17 and from H_3_-18 to H-1 and H_2_-9. In addition, gHMBC correlations from δ_H_ 2.60 (H-1) to δ_C_ 33.7 (C-2), 60.4 (C-5), 74.4 (C-6) and 152.0 (C-10) and from δ_H_ 2.37 (H-5) to 124.2 (C-3), 141.0 (C-4), 74.4 (C-6) and 152.0 (C-10) confirmed H-1 and H-5 as the bridgehead protons and readily identified the perhydroazulene skeleton.

**Table 1:** ^1^H- and ^13^C-NMR data (300 MHz and 75 MHz, CDCl_3_) for dictyol E (**2**).

No.	^13^Cδ (m)	^1^Hδ (m, *J* Hz)	COSY	gHMBC
1	46.0 (d)	2.60 (1H, q, 9.1)	H_2_-2, H-5, H_2_-18	C-2, C-5, C-6, C-10, C-18
2	33.7 (t)	2.51 (1H, m )	H-1, H_b_-2	C-1, C-3, C-4, C-5
		2.22 (1H, dd, 14.8, 7.8)	H-1, H_a_-2, H-3	C-1, C-3, C-4, C-5
3	124.2 (d)	5.34(1H, br s)	H_b_-2, H_3_-17, H-5	C-1, C-2, C-4, C-5, C-17
4	141.0 (s)			
5	60.4 (d)	2.37 (1H, m)	H-1, H_3_-17, H-3, H-6	C-1, C-3, C-4, C-6, C-10
6	74.7 (d)	4.20 (1H, dd, 7.8, 2.0)	H-5, H-7	C-4, C-5, C-7, C-8,
7	48.7 (d)	1.67 (1H, m)	H-6	C-9, C-11, C-12
8	21.6 (t)	1.81 (1H, m)	H_b_-8, H_a_-9	C-6, C-10, C-19
		1.73 (1H, m,)	H_a_-8	C-7, C-11
9	40.6 (t)	2.69 (1H, ddd, 14.8, 4.6, 2.4)	H_b_-9, H_2_-8	C-1, C-7, C-8, C-10, C-18
		2.13 (1H, m )	H_a_-9, H_2_-18	C-8, C-10, C-18
10	152.0 (s)			
11	76.3 (s)			
12	40.9 (t)	1.74 (2H, t, 8.6)	H_2_-13	C-7, C-11, C-13, C-14, C-19
13	23.2 (t)	2.12 (1H, dd, 14.8, 8.6)	H_2_-12, H_b_-13, H-14	C-11, C-12, C-14, C-15
		2.02 (1H, dq, 14.8, 6.9)	H_2_-12, H_b_-13, H-14	C-11, C-12, C-14, C-15
14	124.2 (d)	5.16 (1H, br t, 6.9)	H_2_-13, H_3_-20,	C-12, C-13, C-16, C-20
15	132.0 (s)			
16	25.7 (q)	1.69 (3H, s)	H-14	C-14, C-15, C-20
17	15.9 (q)	1.82 (3H, br s)	H-3, H-5	C-3, C-4, C-5
18	107.4 (t)	4.78 (1H, br s)	H-1	C-1, C-5, C-9, C-10
		4.76 (1H, br s)	H_b_-9	C-1, C-5, C-9, C-10
19	25.3 (q)	1.24 (3H, s)		C-7, C-11, C-12
20	17.5 (q)	1.62 (3H, br s)		C-14, C-15, C-16

Analysis of gHMBC correlations for **2** (Table 1) between δ_H_ 1.74 (H-12) and δ_C_ 76.3 (C-11), δ_C_23.2 (C-13), δ_C_ 124.2 (C-14) and δ_C_ 25.3 (C-19), and between δ_H_ 2.12/2.02 (H-13_a/b_) and δ_C_76.3 (C-11), δ_C_ 40.9 (C-12), δ_C_ 124.2 (C-14) and δ_C_ 25.3 (C-19), confirmed the presence of a 6-methylhept-5-en-2-ol side chain. Furthermore, gHMBC correlations from δ_H_ 1.26 (H_3_-19) to δ_C_ 48.7 (C-7) and from δ_H_ 1.67 (H-7) to δ_C_ 76.3 (C-11) and δ_C_ 40.9 (C-12) allowed the 6-methylhept-5-en-2-ol side chain to be positioned at C-7. Based on these observations, the planar structure of **2** was confirmed as reported [16].

The configuration of the C-3–C-4 double bond must be *Z* in order to form the five-membered ring [26]. The relative stereochemical assignment was confirmed as 1*R*,5*S*,6*R*,7*S* by the positive optical rotation ([α]^21^_D_ +21° CHCl_3_; *c* 0.11) [27] and comparison with literature values [16].

A further two compounds were also isolated from the assemblage, 8α,11-dihydroxypachydictyol A (**3**) and indole-3-carboxaldehyde (**4**). Their spectroscopic data matched those reported in the literature [17,18].

Outlined in Table 2 are the cytotoxic activities of **1**–**4** against a panel of human and mammalian cell lines. From these data there appears to be no obvious SAR, with **1**–**3** having approximately the same activities against the three human tumour cell lines SF-268, MCF-7 and H460. However, the response of compounds **1**–**3** against HT-29, a human colon tumour cell line, and CHO-K1, a Chinese hamster ovary non-tumour cell line, were between two and four-fold less active as compared to those for the three cancer cell lines mentioned above, suggesting some selectivity. Indole-3-carboxaldehyde (**4**) was not active against any of the cell lines.

**Table 2:** Cytotoxicity data [GI_50_ (µM)] for compounds **1**–**4** against the human tumour cell lines SF-268, MCF-7, H460, HT-29, the normal human cell line WI38, and the mammalian cell line CHO-K.

Compound	SF-268 ^a^	MCF-7 ^b^	H460 ^c^	HT-29 ^d^	CHO-K1 ^e^
**1**	25	27	20	61	72
**2**	16	22	17	46	48
**3**	20	38	20	88	103
**4**	NA^f^	NA	NA	NA	NA

^a^ SF-268 Central nervous system-glioblastoma cells; ^b^ MCF-7 Breast-pleural effusion adenocarcinoma cells; ^c^ H460 Lung-large cell carcinoma cells; ^d^ HT-29 Colon-recto-sigmoid colon adenocarcinoma cells; ^e^ CHO-K1 Sub-clone of Chinese hamster ovary cells; ^f^ NA = not active.

## 3. Experimental

### 3.1. General Procedures

General experimental procedures are as described previously [28]. CD spectra were collected on a JASCO J-715 spectropolarimeter with a 0.1 dm cell.

### 3.2. Plant Material

The green/brown algal assemblage of *Halimeda stuposa* (Udoteaceae, Caulerpales) and *Dictyota* sp., (Dictyotaceae, Dictyotales) was collected from the passage between Shaw and Maher Islands, Queensland, at a depth of 7 m, in October 1987. Collection of this material was conducted under the Queensland Fish or Marine Products Permit no. 1780 and the GBRMPA Permit no. 87/293. A voucher specimen (Accession number AQ642006) has been lodged with the Queensland Herbarium.

### 3.3. Bioassay

Cellular bioassays were undertaken as described previously [28].

### 3.4. Extraction and Isolation

Freeze dried plant material was extracted with dichloromethane (CH_2_Cl_2_) (3 × 400 mL) followed by MeOH (3 × 400 mL). The MeOH extract (2.7 g) was then subjected to reversed phase C_18_ flash vacuum chromatography (RP-C18, 0%, 20%, 50%, 70%, 90% and 100% MeOH in H_2_O and 1:1 MeOH:CH_2_Cl_2_). The 50% MeOH fraction was further purified by semi-preparative C_18_ HPLC (4 mL/min, gradient elution from 10% CH_3_CN:H_2_O to 73% CH_3_CN:H_2_O over 14 min through a 250 × 10 mm, 5 μm Phenomenex Luna C_18_ column) to yield the known compound indole-3-carboxyaldehyde (**4**, 0.8 mg, 0.03% dry wt of extract), which had identical physical and spectroscopic properties to those previously published [18].

The active fractions, 90% and 100% MeOH, were each pre-adsorbed onto C_18_, packed into a cartridge, then subjected to C_18_ preparative HPLC (9.5 mL/min, gradient elution from 50% H_2_O:CH_3_CN:0.1% HCO_2_H to 100% CH_3_CN:0.1% HCO_2_H over 40 min, followed by 20 min with 100% CH_3_CN:0.1% HCO_2_H through 250 × 21 mm, 5 μm Phenomenex Luna C_18_ column). The 90% MeOH fraction yielded, 4-hydroxydictyolactone (**1**, 4.9 mg, 0.18% dry wt of extract) as well as 8α,11-dihydroxypachydictyol A (**3**, 10.4 mg, 0.39% dry wt of extract), and the 100% MeOH fraction yielded dictyol E (**2**, 5.5 mg, 0.20% dry wt of extract). The known compounds had identical physical and spectroscopic properties to those previously published [15,16,17].

#### 3.4.1. 4-Hydroxydictyolactone (**1**)

Pale yellow oil. [α]^21^_D_ −87° (CHCl_3_; *c* 0.25); IR cm^−1^: 3436, 2931, 1739, 1455; UV (PDA) nm: 220; CD λ_max_ (Δε) (MeOH; 1.9 × 1*0*^−4^ M) 226 (−38.44), 258 (5.42) nm; ^1^H- (300 MHz, CDCl_3_) and ^13^C- (75 MHz, CDCl_3_) NMR data see Table S1; HRESIMS *m/z* [M+Na]^+^ 341.2103 (calcd for C_20_H_30_O_3_Na 341.2087) [15].

#### 3.4.2. Dictyol E (**2**)

Pale yellow oil. [α]^21^_D_ +21° (CHCl_3_; *c* 0.11); ^1^H- (300 MHz, CDCl_3_) and ^13^C- (75 MHz, CDCl_3_) NMR data (Table 1) were consistent with published values [16].

#### 3.4.3. 8α,11-Dihydroxypachydictyol A (**3**)

Pale yellow oil. ^1^H-NMR and ^13^C-NMR spectral data were consistent with published values [17].

#### 3.4.4. Indole-3-carboxaldehyde (**4**)

Pale yellow solid. ^1^H-NMR and ^13^C-NMR spectral data were consistent with published values [18].

## 4. Conclusions

Four compounds, the xenicane diterpene 4-hydroxydictyolactone (**1**), and the diterpenes dictyol E (**2**), 8α,11-dihydroxypachydictyol A (**3**) and indole-3-carboxaldehyde (**4**), were isolated from an assemblage of *Halimeda stuposa* and a *Dictyota* sp. Although there are many reports on the isolation of xenicane diterpenes from algae of the genera *Dictyota* sp. [12,29,30,31,32,33,34], *Pachydictyon* sp. [35,36,37], *Glossophora* sp. [38] and *Dilophus* sp. [15,16,39], with the latter three genera now recognized as *Dictyota* species [40], and of pachydictyane diterpenes from algae of the genera *Dictyota* sp. [41], *Sargassum* sp. [42], *Glossophora* sp. [26] and *Cystoseira* sp. [43], there are very few that discuss their cytotoxic properties (xenicanes: [12,21,29]; pachydictyanes: [21,42,43,44]). The bioactivity results and the updated spectroscopic data presented in the current work clearly show that more detailed and concerted investigations of these two classes of diterpenes are warranted.

## Supplementary Materials

Supplementary materials can be accessed at: http://www.mdpi.com/1420-3049/17/3/2929/s1.
