# Supplementary material for: Update of Spectroscopic Data for 4-Hydroxydictyolactone and Dictyol E Isolated from a Halimeda stuposa — Dictyota sp. Assemblage †
Source: Molecules. 2012 Mar 8;17(3):2929–38. doi: 10.3390/molecules17032929 (PMC6268996; doi:10.3390/molecules17032929)
Supplement: Supplementary file 1 [file molecules-17-02929-s001.pdf]

Supporting Information for

Update of spectroscopic data for 4-hydroxyldictyolactone and dictyol E isolated from a *Halimeda stuposa* - *Dictyota* sp. Assemblage

Simon P. B. Ovenden,[†] Jonathan L. Nielson,[‡] Catherine H. Liptrot,[§] Richard H. Willis,[⊥] Dianne M. Tapiolas,[⊥] Anthony D. Wright[‖] and Cherie A. Motti[⊥,*]

Australian Institute of Marine Science, PMB no. 3, Townsville MC, Townsville, 4810, Australia

Current address: Defence Science & Technology Organisation, 506 Lorimer St. Fishermans Bend, Victoria, 3207, Australia

Current address: ACD Labs UK, Building A, Trinity Court, Wokingham Road, Bracknell, Berkshire, RG42 1PL, England

Current address: James Cook University, Townsville, QLD 4811, Australia.

Current address: University of Hawaii at Hilo, College of Pharmacy, 34 Rainbow Drive, Hilo, Hawaii 96720, USA.

[*] To whom correspondence should be addressed: Tel.: +61 7 4753 4143. Fax.: +61 7 4772 5852. Email: c.motti@aims.gov.au. [†]*Defence Science & Technology Organisation*, [‡]*ACD Labs UK*, [§]*James Cook University*, [⊥]*Australian Institute of Marine Science*, [‖]*University of Hawaii at Hilo.*

Table of Contents.

Page No

Figure S1. ^{1}H NMR spectrum (300 MHz) of 4-hydroxydictyolactone (**1**) in $CDCl_3$........ 3

Figure S2. ^{13}C NMR spectrum (75 MHz) of 4-hydroxydictyolactone (**1**) in $CDCl_3$........4

Figure S3. COSY spectrum (300 MHz) of 4-hydroxydictyolactone (**1**) in $CDCl_3$........ 5

Figure S4. HSQC spectrum (300 MHz) of 4-hydroxydictyolactone (**1**) in $CDCl_3$. 6

Figure S5. HMBC spectrum (300 MHz) of 4-hydroxydictyolactone (**1**) in $CDCl_3$........7

Figure S6. ^{1}H NMR spectrum (300 MHz) of dictyol E (**2**) in $CDCl_3$........ 8

Figure S7. COSY spectrum (300 MHz) of dictyol E (**2**) in $CDCl_3$........ 9

Figure S8. HSQC spectrum (300 MHz) of dictyol E (**2**) in $CDCl_3$........ 10

Figure S9. HMBC spectrum (300 MHz) of dictyol E (**2**) in $CDCl_3$........ 11

Table S1. ^{1}H and ^{13}C NMR data (300 MHz and 75 MHz, $CDCl_3$) for the *trans*- and *cis*-conformers of 4-hydroxydictyolactone (**1**)........12

Table S2. Comparison of calculated dihedral angles from the 16 possible stereoisomers of the H-3 – C-20 *trans*-conformers and the corresponding *cis*-conformers of **1** with observed ^{1}H-^{1}H couplings from the ^{1}H NMR. * indicates those stereoisomers/conformers that match the ^{1}H NMR data........13

S1. ^{1}H NMR of **1**

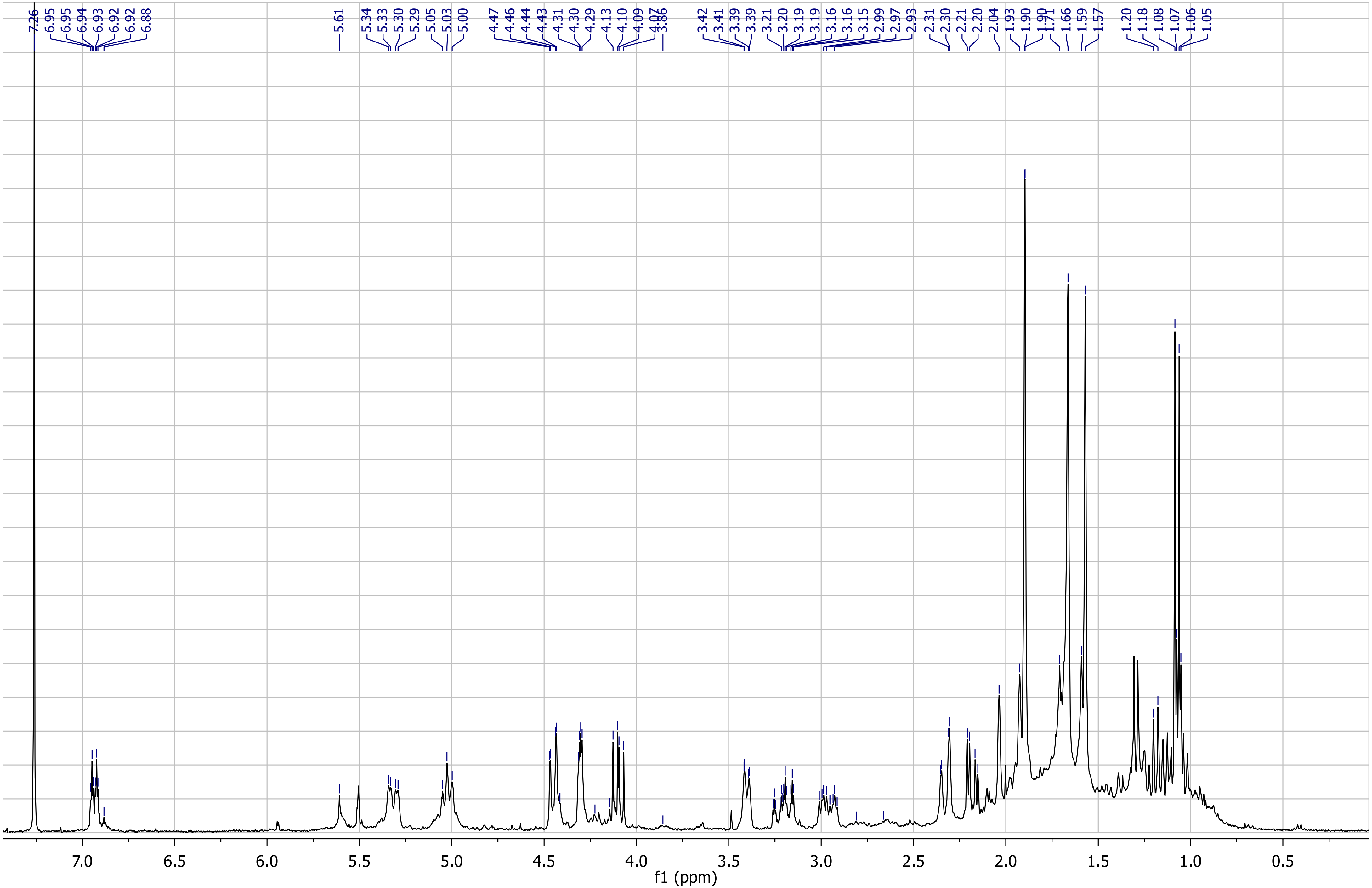

7.26
6.95
6.95
6.94
6.93
6.92
6.92
6.88
5.61
5.34
5.33
5.30
5.29
5.05
5.03
5.00
4.47
4.46
4.44
4.43
4.31
4.30
4.29
4.13
4.10
4.09
4.07
3.86
3.42
3.41
3.39
3.39
3.21
3.20
3.19
3.19
3.16
3.16
3.15
2.99
2.97
2.93
2.31
2.30
2.21
2.20
2.04
1.93
1.90
1.90
1.71
1.66
1.59
1.57
1.20
1.18
1.08
1.07
1.06
1.05
7.0
6.5
6.0
5.5
5.0
4.5
4.0
3.5
3.0
2.5
2.0
1.5
1.0
0.5
f1 (ppm)

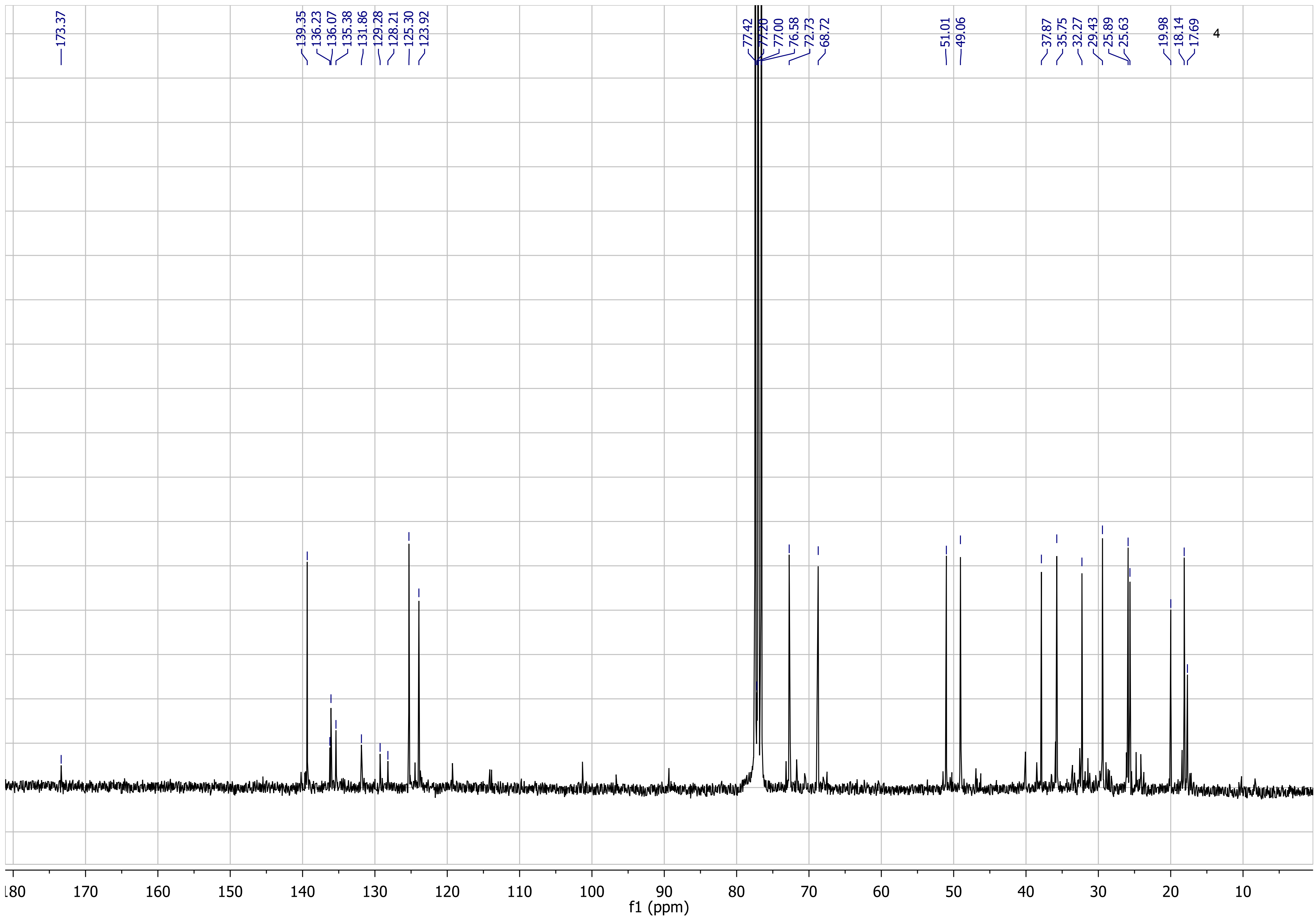

173.37
139.35
136.23
136.07
135.38
131.86
129.28
128.21
125.30
123.92
77.42
77.20
77.00
76.58
72.73
68.72
51.01
49.06
37.87
35.75
32.27
29.43
25.89
25.63
19.98
18.14
17.69
4
180
170
160
150
140
130
120
110
100
90
80
70
60
50
40
30
20
10
f1 (ppm)

S3. COSY NMR of **1**

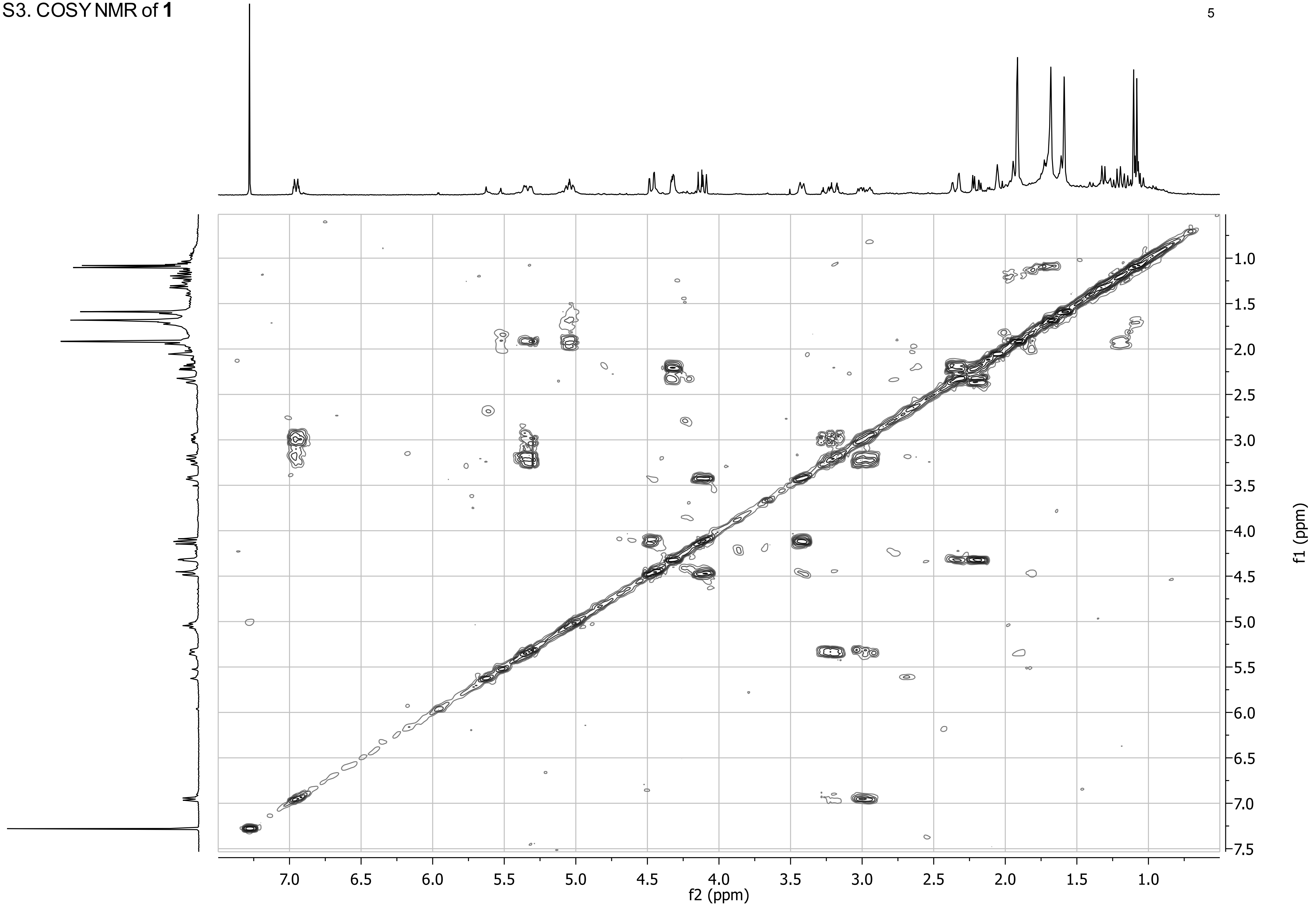

S4. HSQC NMR of **1**

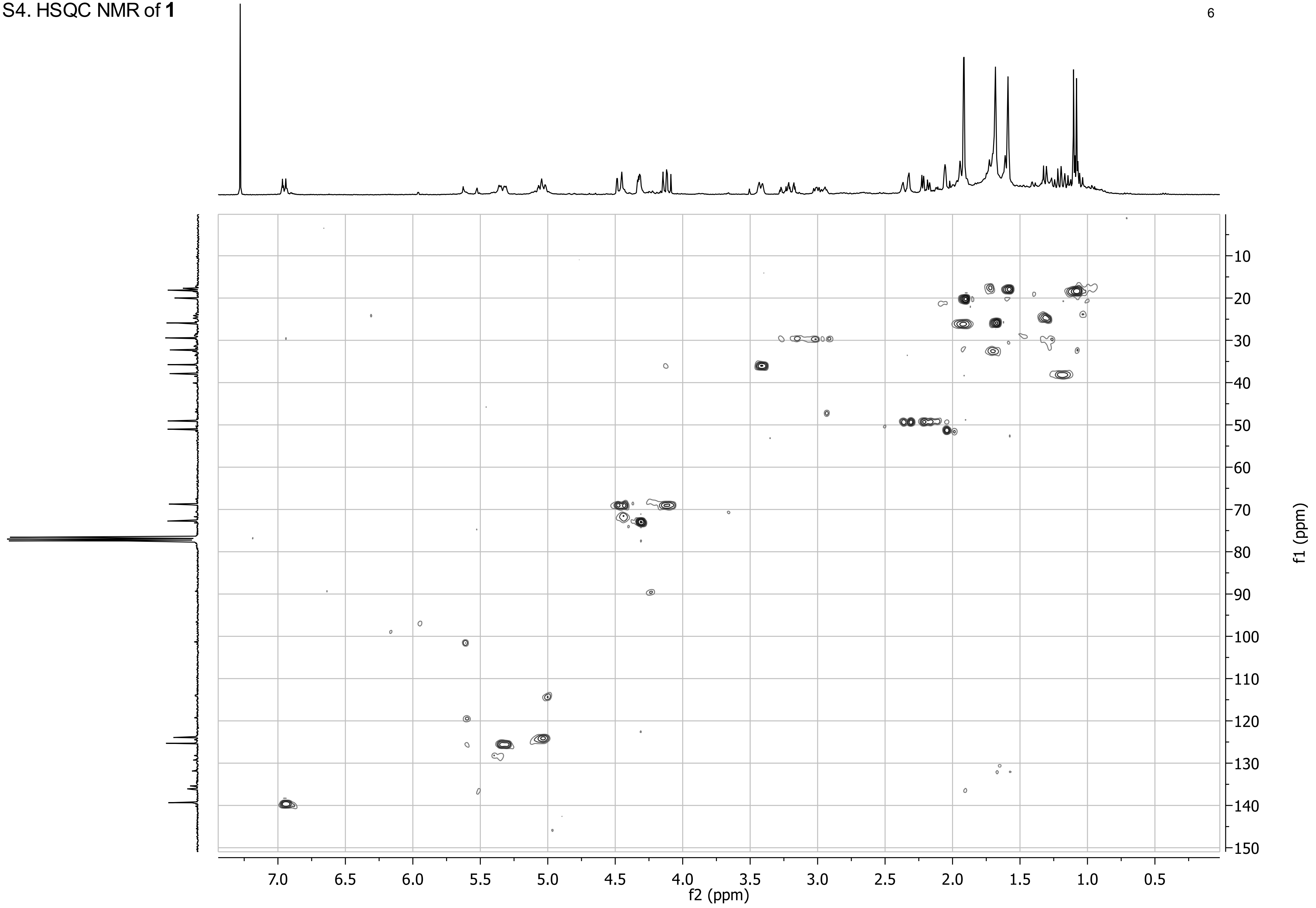

S5. HMBC NMR of **1**

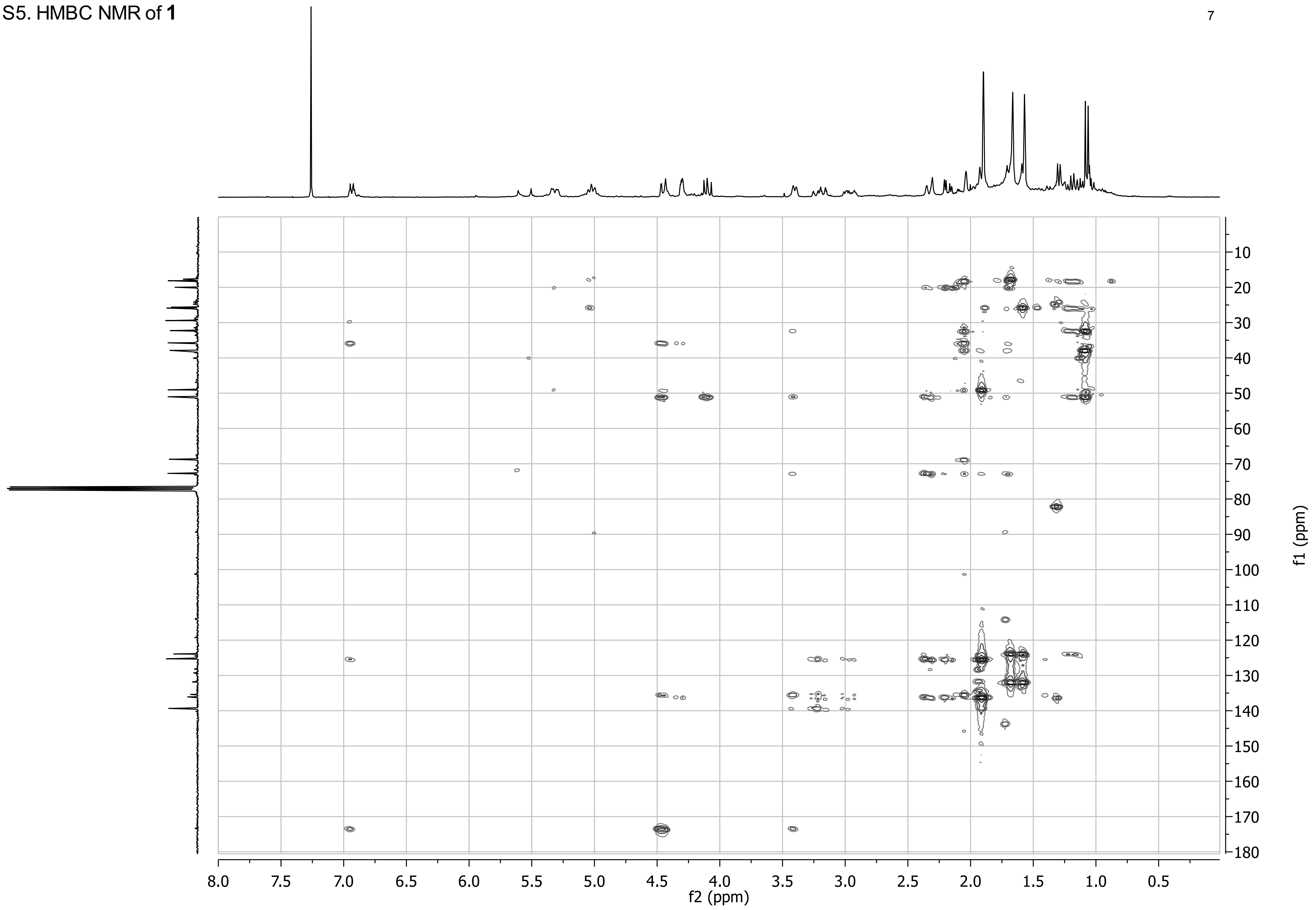

S6. ^{1}H NMR of **2**

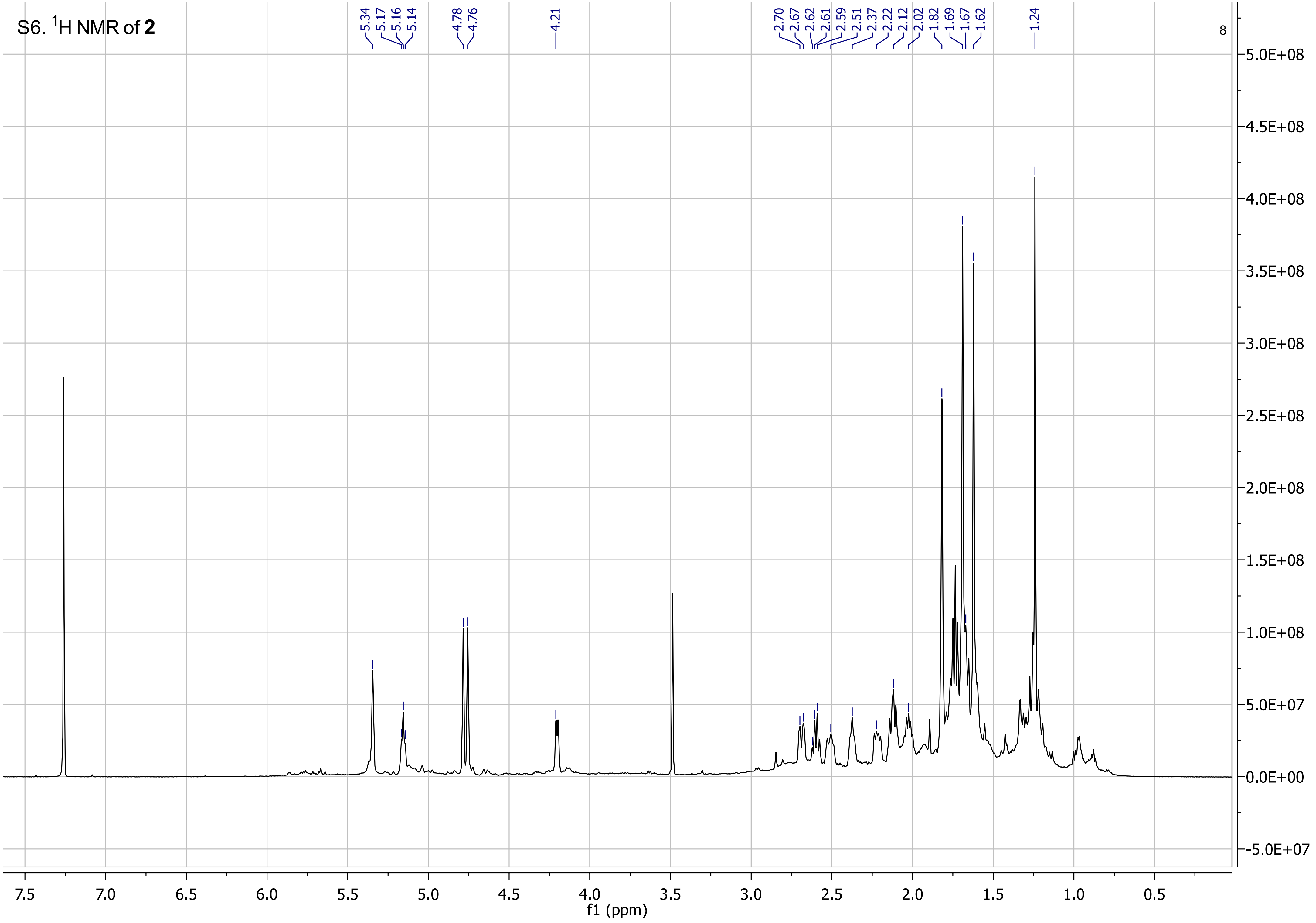

5.34
5.17
5.16
5.14
4.78
4.76
4.21
2.70
2.67
2.62
2.61
2.59
2.51
2.37
2.22
2.12
2.02
1.82
1.69
1.67
1.62
1.24
f1 (ppm)

S7. COSY NMR of **2**

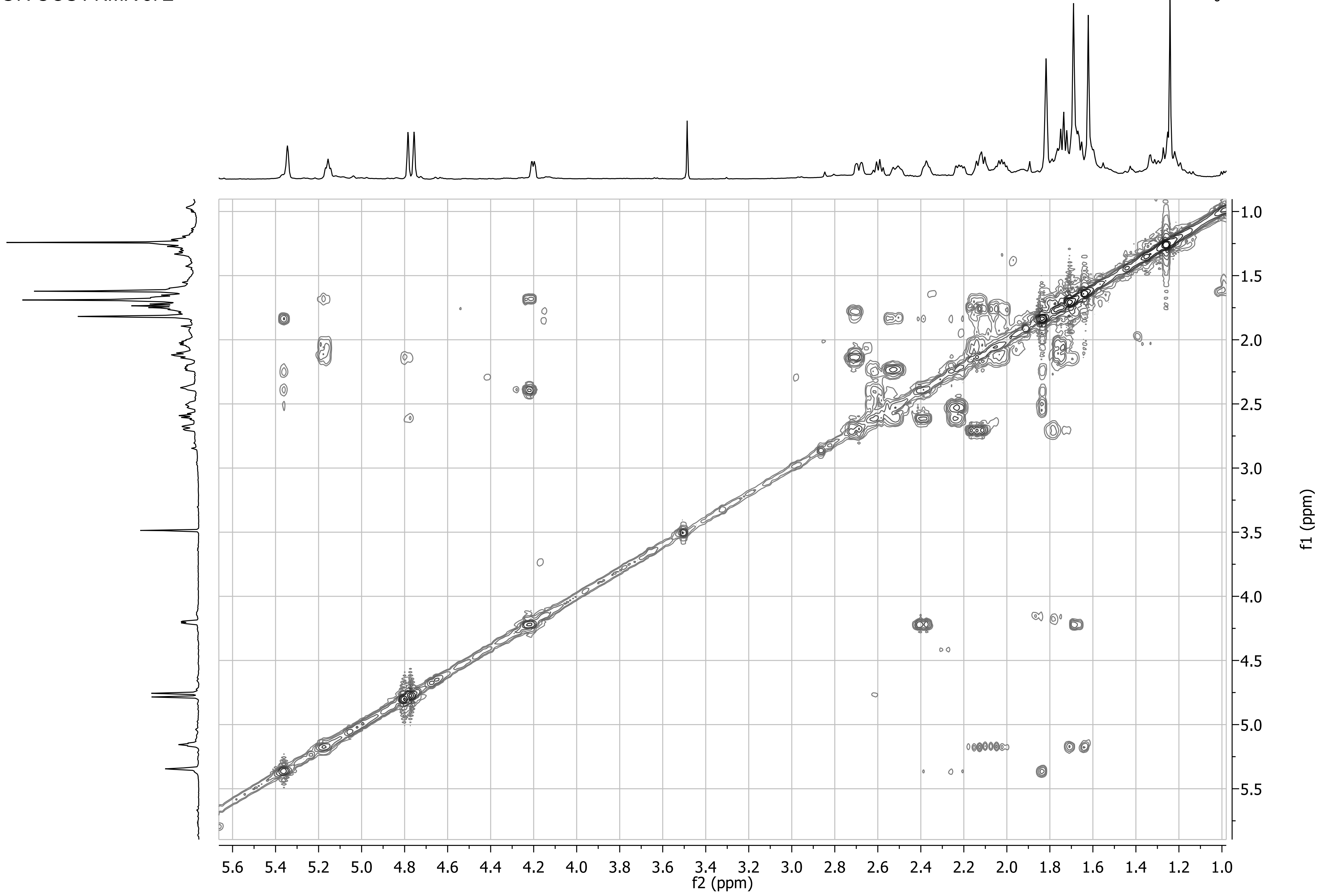

S8. HSQC NMR of **2**

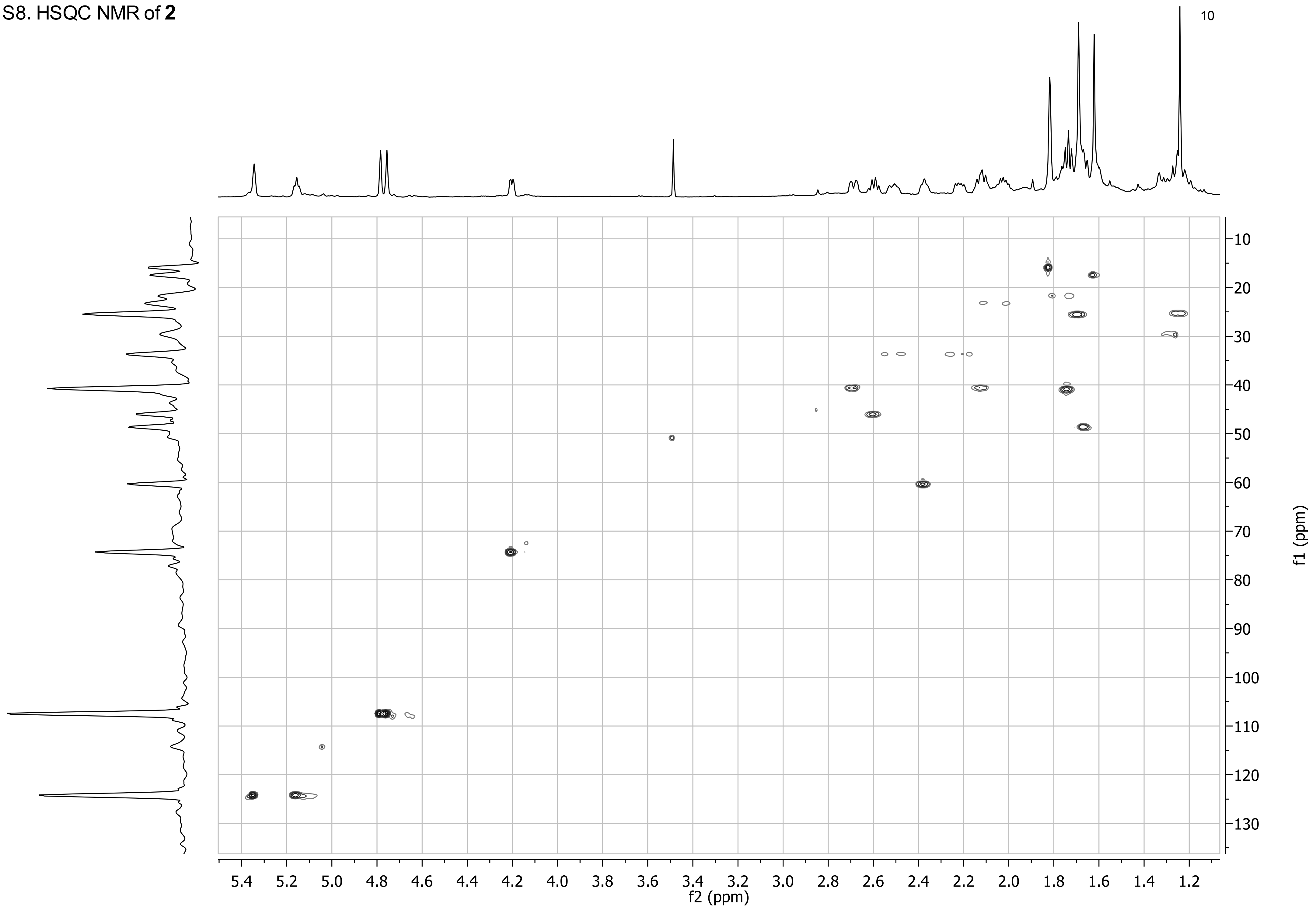

S9. HMBC NMR of **2**

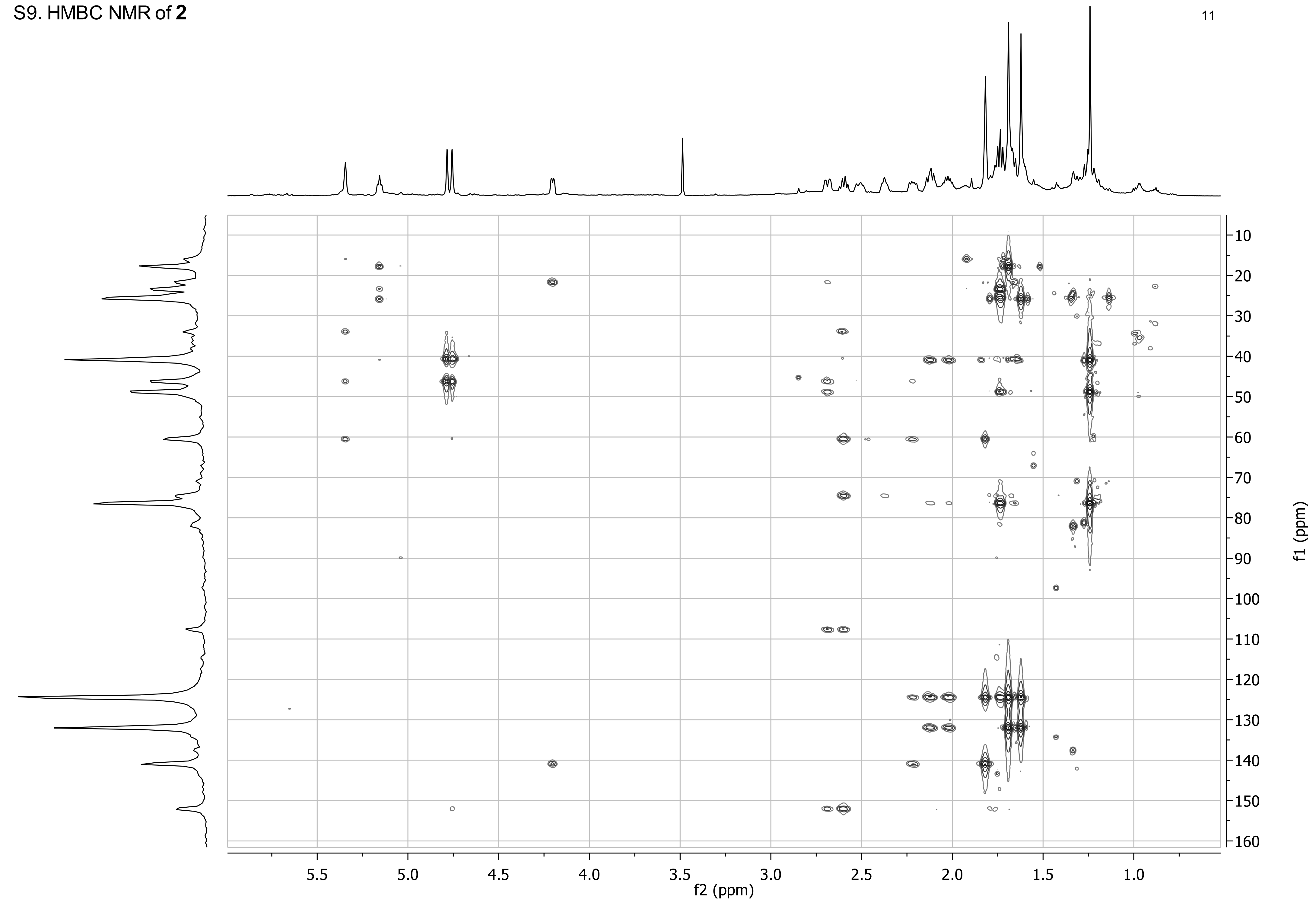

Table S1. ^{1}H and ^{13}C NMR data (300 MHz and 75 MHz, $CDCl_3$) for the *trans*- and *cis*-conformers of 4-hydroxydictyolactone (**1**).

***Trans*-conformer (major compound)**					***Cis*-conformer (minor compound)**	
**No.**	**^{13}C δ (m)**	**^{1}H δ (m, *J* Hz)**	**COSY**	**gHMBC**	**^{13}C δ (m)**	**^{1}H δ (m, *J* Hz)**
1	135.4 (s)					
2	35.8 (d)	3.40 (1H, dd, 7.8, 1.2)	H_2-18	C-1, C-3, C-4, C-9, C-10, C-19	35.5 (d)	3.86 (m)
3	51.0 (d)	2.04 (1H, br s)		C-1, C-2, C-4, C-5, C-10, C-11, C-17, C-18	50.9	1.71 (br s)
4	72.7 (d)	4.30 (1H, dd, 4.2, 2.2)	H_a-5, H_b-5	C-2, C-6	68.1 (d)	4.22 (m)
5	49.1 (t)	2.33 (1H, dd, 13.0, 2.2)	H-4, H_b-5	C-3, C-4, C-6, C-7, C-20	ND	2.81 (m)
		2.18 (1H, dd, 13.0, 4.2)	H-4, H_a-5	C-3, C-4, C-6, C-7, C-20		2.32 (m)
6	136.1 (s)					
7	125.3 (d)	5.32 (1H, ddq, 11.4, 4.2, 0.7)	H_a-8, H_b-8, H_3-20	C-5, C-20	125.4 (d)	5.61 (m)
8	29.4 (t)	3.20 (1H, dddd, 17.5, 11.4, 2.2, 2.2)	H-7, H_b-8, H-9	C-1, C-6, C-7, C-9	ND	3.21 (m)
		2.98 (1H, ddd, 17.5, 7.4, 4.2)	H-7, H_a-8, H-9	C-1, C-6, C-7, C-9		2.66 (m)
9	139.5 (d)	6.94 (1H, dt, 7.4, 2.2)	H_a-8, H_b-8	C-2, C-7, C-8, C-19	139.8 (d)	6.88 (dt, 8.0, 3.3)
10	32.3 (d)	1.71 (1H, m)	H_3-17	C-2, C-3, C-4, C-11, C-12, C-17	32.6 (d)	1.72 (m)
11	37.9 (t)	1.19 (2H, m)	H_2-12		ND	
12	25.9 (t)	1.93 (2H, m)	H_2-11, H-13	C-14	ND	
13	123.9 (d)	5.02 (1H, t hept, 7.2, 1.4)	H_2-12, H_3-15, H_3-16	C-15, C-16	124.3 (d)	5.07 (1H, t hept, 7.2, 1.4)
14	131.9 (s)					
15	17.7 (q)	1.57 (3H, s)	H-13	C-13, C-14, C-16	25.7 (q)	1.59 (3H, s)
16	25.6 (q)	1.66 (3H, s)	H-13	C-13, C-14, C-15	17.4 (q)	1.74 (3H, s)
17	18.1 (q)	1.07 (3H, d, 6.7)	H-10	C-3, C-10, C-11	18.1 (q)	1.06 (d, 6.7)
18	68.7 (t)	4.45 (1H, dd, 9.7, 1.2)	H-2, H_b-18	C-1, C-2, C-3, C-19	68.9 (t)	4.41 (dd, 9.6, 1.6)
		4.10 (1H, dd, 9.7, 7.8)	H-2, H_a-18	C-3		4.14 (dd, 9.6, 7.3)
19	173.3 (s)				ND	
20	20.0 (q)	1.90 (3H, br d, 0.7)	H-7	C-5, C-6, C-7	25.7 (q)	1.59 (br s)

ND = Not detected

Table S2. Comparison of calculated dihedral angles from the 16 possible stereoisomers of the H-3 – C-20 *trans*-conformers and the corresponding *cis*-conformers of **1** with observed ^{1}H-^{1}H couplings from the ^{1}H NMR. * indicates those stereoisomers/conformers that match the ^{1}H NMR data.

C-2, C-3, C-4, C-10 configuration	^{1}H-^{1}H correlation	Calculated dihedral angle Φ	Calculated *J* (Hz)	Relevant observed ^{1}H-^{1}H couplings
SSSS trans	H2-C2-C3-H3	-94	0.91	No observed coupling: H-2 couples to H_2-18 only
	H3-C3-C4-H4	154	8.94	NA
	H3-C3-C10-H10	60	2.72	No observed coupling: H-3 (br s), H-10 couples to H_3-17 only.
SSSS cis	H2-C2-C3-H3	138	6.71	NA
	H3-C3-C4-H4	-47	4.94	NA
	H3-C3-C10-H10	166	10.61	NA
SSSR trans	H2-C2-C3-H3	-99	1.18	No observed coupling: H-2 couples to H_2-18 only
	H3-C3-C4-H4	158	9.30	NA
	H3-C3-C10-H10	-90	0.80	No observed coupling: H-3 (br s), H-10 couples to H_3-17 only.
SSSR cis	H2-C2-C3-H3	139	6.89	NA
	H3-C3-C4-H4	-48	4.81	NA
	H3-C3-C10-H10	-176	11.14	NA
**SSRR trans*	H2-C2-C3-H3	-102	1.40	No observed coupling: H-2 couples to H_2-18 only
	H3-C3-C4-H4	-87	0.80	No observed coupling: H-3 (br s)
	H3-C3-C10-H10	-85	0.80	No observed coupling: H-3 (br s), H-10 couples to H_3-17 only.
SSRR cis	H2-C2-C3-H3	134	6.01	NA
	H3-C3-C4-H4	80	1.31	No observed coupling: H-3 (br s)
	H3-C3-C10-H10	-175	11.11	NA
SRRR trans	H2-C2-C3-H3	-59	2.85	No observed coupling: H-2 couples to H_2-18 only
	H3-C3-C4-H4	-127	5.25	NA
	H3-C3-C10-H10	70	1.60	No observed coupling: H-3 (br s), H-10 couples to H_3-17 only.
SRRR cis	H2-C2-C3-H3	104	1.58	No observed coupling: H-2 couples to H_2-18 only
	H3-C3-C4-H4	62	3.02	NA
	H3-C3-C10-H10	-126	4.61	NA
**SSRS trans*	H2-C2-C3-H3	-94	0.91	No observed coupling: H-2 couples to H_2-18 only
	H3-C3-C4-H4	-89	0.87	No observed coupling: H-3 (br s)

	H3-C3-C10-H10	70	1.60	No observed coupling: H-3 (br s), H-10 couples to H_3-17 only.
SSRS cis	H2-C2-C3-H3	137	6.54	NA
	H3-C3-C4-H4	74	1.76	No observed coupling: H-3 (br s)
	H3-C3-C10-H10	-174	11.08	NA
SRRS trans	H2-C2-C3-H3	-68	1.79	No observed coupling: H-2 couples to H_2-18 only
	H3-C3-C4-H4	-125	4.94	NA
	H3-C3-C10-H10	-79	0.97	No observed coupling: H-3 (br s), H-10 couples to H_3-17 only.
SRRS cis	H2-C2-C3-H3	68	1.79	No observed coupling: H-2 couples to H_2-18 only
	H3-C3-C4-H4	155	7.64	NA
	H3-C3-C10-H10	88	0.78	No observed coupling: H-3 (br s), H-10 couples to H_3-17 only.
SRSR-trans	H2-C2-C3-H3	114	2.74	No observed coupling: H-2 couples to H_2-18 only
	H3-C3-C4-H4	-48	4.44	NA
	H3-C3-C10-H10	68	1.79	No observed coupling: H-3 (br s), H-10 couples to H_3-17 only.
SRSR-cis	H2-C2-C3-H3	71	1.51	
	H3-C3-C4-H4	34		NA
	H3-C3-C10-H10	-82	0.86	No observed coupling: H-3 (br s), H-10 couples to H_3-17 only.
SRSS trans	H2-C2-C3-H3	-47	4.59	NA
	H3-C3-C4-H4	110	2.75	No observed coupling: H-3 (br s)
	H3-C3-C10-H10	-60	2.72	No observed coupling: H-3 (br s), H-10 couples to H_3-17 only.
SRSS cis	H2-C2-C3-H3	70	1.60	No observed coupling: H-2 couples to H_2-18 only
	H3-C3-C4-H4	35	4.70	NA
	H3-C3-C10-H10	67	1.89	No observed coupling: H-3 (br s), H-10 couples to H_3-17 only.
RSRS trans	H2-C2-C3-H3	14	8.84	NA
	H3-C3-C4-H4	94	0.89	No observed coupling: H-3 (br s)
	H3-C3-C10-H10	75	1.20	No observed coupling: H-3 (br s), H-10 couples to H_3-17 only.
RSRS cis	H2-C2-C3-H3	-109	2.10	No observed coupling: H-2 couples to H_2-18 only
	H3-C3-C4-H4	68	2.34	No observed coupling: H-3 (br s)
	H3-C3-C10-H10	-157	9.67	NA
**RRSR trans*	H2-C2-C3-H3	102	1.40	No observed coupling: H-2 couples to H_2-18 only
	H3-C3-C4-H4	87	0.80	No observed coupling: H-3 (br s)
	H3-C3-C10-H10	84	0.81	No observed coupling: H-3 (br s), H-10 couples to H_3-17 only.
RRSR cis	H2-C2-C3-H3	-139	6.89	NA
	H3-C3-C4-H4	-71	2.03	No observed coupling: H-3 (br s)

	H3-C3-C10-H10	-178	11.18	NA
RSSR trans	H2-C2-C3-H3	58	2.98	No observed coupling: H-2 couples to H_2-18 only
	H3-C3-C4-H4	121	4.32	NA
	H3-C3-C10-H10	-60	2.72	No observed coupling: H-3 (br s), H-10 couples to H_3-17 only.
RSSR cis	H2-C2-C3-H3	-108	1.99	No observed coupling: H-2 couples to H_2-18 only
	H3-C3-C4-H4	-51	4.42	NA
	H3-C3-C10-H10	-173	11.04	NA
RSRR trans	H2-C2-C3-H3	48	4.44	NA
	H3-C3-C4-H4	-106	2.26	No observed coupling: H-3 (br s)
	H3-C3-C10-H10	54	3.54	NA
RSRR cis	H2-C2-C3-H3	-104	1.58	No observed coupling: H-2 couples to H_2-18 only
	H3-C3-C4-H4	63	2.90	No observed coupling: H-3 (br s)
	H3-C3-C10-H10	-142	7.40	NA
RRRR trans	H2-C2-C3-H3	99	1.18	No observed coupling: H-2 couples to H_2-18 only
	H3-C3-C4-H4	-154	8.94	NA
	H3-C3-C10-H10	82	0.86	No observed coupling: H-3 (br s), H-10 couples to H_3-17 only.
RRRR cis	H2-C2-C3-H3	-146	8.07	NA
	H3-C3-C4-H4	58	3.51	NA
	H3-C3-C10-H10	-134	6.01	NA
RRRS trans	H2-C2-C3-H3	97	1.05	No observed coupling: H-2 couples to H_2-18 only
	H3-C3-C4-H4	-158	9.30	NA
	H3-C3-C10-H10	-54	3.54	NA
RRRS cis	H2-C2-C3-H3	-155	9.41	NA
	H3-C3-C4-H4	71	2.03	No observed coupling: H-3 (br s)
	H3-C3-C10-H10	80	0.93	No observed coupling: H-3 (br s), H-10 couples to H_3-17 only.
**RRSS trans*	H2-C2-C3-H3	102	1.40	No observed coupling: H-2 couples to H_2-18 only
	H3-C3-C4-H4	86	0.77	No observed coupling: H-3 (br s)
	H3-C3-C10-H10	86	0.79	No observed coupling: H-3 (br s), H-10 couples to H_3-17 only.
RRSS cis	H2-C2-C3-H3	-135	6.18	NA
	H3-C3-C4-H4	-74	1.76	No observed coupling: H-3 (br s)
	H3-C3-C10-H10	-178	11.18	NA
RSSS trans	H2-C2-C3-H3	49	4.28	NA
	H3-C3-C4-H4	121	4.32	NA

	H3-C3-C10-H10	173	11.04	NA
RSSS cis	H2-C2-C3-H3	-111	2.35	No observed coupling: H-2 couples to H_2-18 only
	H3-C3-C4-H4	-51	4.42	NA
	H3-C3-C10-H10	-161	10.13	NA
